# Simultaneous quantification of the most common and proteolytic *Pseudomonas* species in raw milk by multiplex qPCR

**DOI:** 10.1007/s00253-021-11109-0

**Published:** 2021-02-01

**Authors:** Christopher Maier, Katharina Hofmann, Christopher Huptas, Siegfried Scherer, Mareike Wenning, Genia Lücking

**Affiliations:** 1 ZIEL Institute for Food and Health, Wissenschaftszentrum Weihenstephan, Technische Universität München, Weihenstephaner Berg 1, 85354 Freising, Germany; 2 Lehrstuhl für Mikrobielle Ökologie, Wissenschaftszentrum Weihenstephan, Technische Universität München, Weihenstephaner Berg 3, 85354 Freising, Germany; 3 Bavarian Health and Food Safety Authority (LGL), Veterinärstr. 2, 85764 Oberschleißheim, Germany

**Keywords:** Multiplex quantitative PCR, *Pseudomonas*, Proteolytic milk spoilage, *aprX*

## Abstract

**Abstract:**

The heat-stable peptidase AprX, secreted by psychrotolerant *Pseudomonas* species in raw milk, is a major cause of destabilization and premature spoilage of ultra-high temperature (UHT) milk and milk products. To enable rapid detection and quantification of seven frequent and proteolytic *Pseudomonas* species (*P. proteolytica*, *P. gessardii*, *P. lactis*, *P. fluorescens*, *P. protegens*, *P. lundensis*, and *P. fragi*) in raw milk, we developed two triplex qPCR assays taking into account species-dependent differences in AprX activity. Besides five species-specific hydrolysis probes, targeting the *aprX* gene, a universal *rpoB* probe was included in the assay to determine the total *Pseudomonas* counts. For all six probes, linear regression lines between *C*_q_ value and target DNA concentration were obtained in singleplex as well as in multiplex approaches, yielding *R*^2^ values of > 0.975 and amplification efficiencies of 85–97%. Moreover, high specificity was determined using genomic DNA of 75 *Pseudomonas* strains, assigned to 57 species, and 40 other bacterial species as templates in the qPCR. Quantification of the target species and total *Pseudomonas* counts resulted in linear detection ranges of approx. 10^3^–10^7^ cfu/ml, which correspond well to common *Pseudomonas* counts in raw milk. Application of the assay using 60 raw milk samples from different dairies showed good agreement of total *Pseudomonas* counts calculated by qPCR with cell counts derived from cultivation. Furthermore, a remarkably high variability regarding the species composition was observed for each milk sample, whereby *P. lundensis* and *P. proteolytica*/*P. gessardii* were the predominant species detected.

**Key points:**

*• Multiplex qPCR for quantification of seven proteolytic Pseudomonas species and total Pseudomonas counts in raw milk*

*• High specificity and sensitivity via hydrolysis probes against aprX and rpoB*

*• Rapid method to determine Pseudomonas contamination in raw milk and predict spoilage potential*

**Supplementary Information:**

The online version contains supplementary material available at 10.1007/s00253-021-11109-0.

## Introduction

Premature spoilage of ultra-high temperature (UHT) milk and milk products due to microbial extracellular enzymes is challenging for the dairy industry, both from an economic and a technical point of view (Hantsis-Zacharov and Halpern 2007; Marchand et al. 2009b; Stoeckel et al. 2016a, b; von Neubeck et al. 2016). Cold storage of raw milk before processing favors the growth of psychrotolerant bacteria, especially *Pseudomonas*, which soon dominate the microbiota (Lafarge et al. 2004; De Jonghe et al. 2011; von Neubeck et al. 2015). Several *Pseudomonas* species produce the extracellular, caseinolytic peptidase AprX, which is heat stable and remains partly active even after UHT treatment. Residual AprX activity can then cause negative effects in milk, such as off-flavors, particle formation, fat separation, or age gelation, all leading to instability and shelf life reduction of processed dairy products (McKellar 1981; Sørhaug and Stepaniak 1997; Matéos et al. 2015; Stoeckel et al. 2016a; Marchand et al. 2017).

The alkaline zinc-metallopeptidase AprX, belonging to the serralysin protease family, has a molecular weight of 45–50 kDa and is encoded by the polycistronic *aprX-lipA2* operon (Schokker and van Boekel 1997; Woods et al. 2001; Marchand et al. 2009b). For many *aprX*-possessing *Pseudomonas* species, this operon additionally includes genes coding for a peptidase inhibitor (AprI), an ABC-transport system (AprDEF), two putative autotransporters (PrtA and PrtB), and a lipase (LipA) (Duong et al. 2001; Woods et al. 2001; Maier et al. 2020). Several studies revealed a high variability of milk-associated *Pseudomonas* species and strains regarding their proteolytic potential, which has been proposed to be due to different gene expression and regulation mechanisms (Dufour et al. 2008; Marchand et al. 2009b; Bagliniere et al. 2012; von Neubeck et al. 2015; Caldera et al. 2016). However, genetic variations also seem to play a role, as *aprX* gene sequences of *Pseudomonas* spp. isolated from raw milk were shown to be very heterogeneous (Marchand et al. 2009b). Moreover, different *aprX-lipA2* operon structures were identified in the genus and a correlation between the type of operon organization and the proteolytic potential of pseudomonads was observed (Maier et al. 2020). Regarding the occurrence in raw milk, *Pseudomonas proteolytica*, *Pseudomonas lundensis*, *Pseudomonas lactis*, *Pseudomonas fragi*, *Pseudomonas protegens*, *Pseudomonas gessardii*, and *Pseudomonas fluorescens* were found to be the most frequent species, revealing various proteolytic capacities. While strains of *P. proteolytica*, *P. lactis*, *P. protegens*, *P. gessardii*, and *P. fluorescens* exhibited mainly high proteolytic activity, isolates of *P. lundensis* or *P. fragi* had middle or low proteolytic potential (Marchand et al. 2009a; De Jonghe et al. 2011; Baur et al. 2015; von Neubeck et al. 2015; Caldera et al. 2016; Glück et al. 2016; Maier et al. 2020).

Sensitive and rapid applications for determination of milk-spoiling *Pseudomonas* strains or AprX amounts are required to control raw milk quality and avoid deterioration of processed dairy products. Time-consuming culturing on selective media is not suitable to predict the spoilage potential of raw milk samples prior to processing. Regarding molecular methods, only a few immunological assays with monoclonal antibodies directed against single AprX proteins of specific *Pseudomonas* strains have been developed, which are not appropriate for a broader application in raw milk containing multiple species (Birkeland et al. 1985; Clements et al. 1990; Matta et al. 1997). Moreover, PCR-based approaches have been performed using *aprX* as a target gene to indirectly detect the spoilage potential (Martins et al. 2005; Marchand et al. 2009b; Machado et al. 2013). However, these methods were applied in pasteurized, reconstituted, or sterilized milk and are not sensitive enough to be used in raw milk, having a lower detection limit of, e.g., 10^7^ colony-forming units (cfu) per ml (Machado et al. 2015). Also, most former molecular assays focused on the *aprX* or peptidase detection of *P. fluorescens* strains, neglecting other common milk-spoiling species, such as members of the *P. gessardii* and *P. fragi* subgroup*.*

Consequently, until now, there is no genetic method to discriminate between distinct *Pseudomonas* species with various proteolytic activity present in raw milk. Thus, the aim of this study was to develop a species-specific multiplex qPCR assay, able to quantify seven of the most frequent and proteolytic *Pseudomonas* species in raw milk as well as the total *Pseudomonas* counts. Overall, two triplex assays were established using species-specific probes, targeting *aprX* gene sequences, and one universal *rpoB* probe, directed against all members of the genus.

## Material and methods

### Bacterial strains and growth conditions

Bacterial strains used in this study are listed in the Supplementary Table S1. In total, 75 strains of 57 *Pseudomonas* species and isolates of 40 other bacterial species, belonging to 25 different genera, were chosen. Among them, 61 strains originated from raw milk, 18 from environmental samples, 9 from milk or semi-finished milk products, 8 from water, 8 from soil, 4 from food environments, and 4 from human samples. For cultivation, most bacterial strains were grown under aerobic conditions on TS-agar (Carl Roth GmbH, Karlsruhe, Germany) at 30 °C for 24–96 h. *Bifidobacterium longum* was cultivated under anaerobic conditions at 37 °C on TOS-agar (Merck KGaA, Darmstadt, Germany). Members of *Lactobacillus*, *Leuconostoc*, and *Lactococcus* were grown under anaerobic conditions at 30 °C on APT-agar for 48 h (Merck KGaA, Darmstadt, Germany). Overnight cultures of *Pseudomonas* spp. were performed by inoculating 4 ml tryptic soy broth (TSB, Merck KGaA, Darmstadt, Germany) with cell material from one colony and incubated at 30 °C and 150 rpm for 16 h. Cell counts were determined on TS-agar (total cell count) as well as on selective CFC-agar (*Pseudomonas* cell count, Merck KGaA, Darmstadt, Germany) after incubation at 30 °C for 24 h.

### Raw milk samples

For spiking experiments, fresh raw milk was obtained from a test farm of TUM (Forschungsstation Veitshof, Freising, Germany) and stored at − 20 °C until use, to ensure constant experimental conditions. For validation of the qPCR assay, 60 raw milk samples from 13 different dairies located all over Germany were analyzed. All samples were shipped refrigerated for 1–3 days. Total and *Pseudomonas* cell counts of raw milk samples were determined immediately after receipt, and the remaining samples were stored at − 20 °C until further processing.

### Bacterial DNA extraction from raw milk samples

Bacterial DNA was extracted from raw milk samples using the DNeasy® PowerFood® Microbial Kit (Qiagen N.V., Hilden, Germany) combined with an EDTA pre-treatment. In brief, raw milk samples of 7.2 ml (4 × 1.8 ml) were centrifuged (2 min, 16000*g*, room temperature (RT)), and the supernatants and the covering fat layers were removed. The remaining pellets were then resuspended and united in a total of 1 ml ¼ Ringer’s solution (Merck KGaA, Darmstadt, Germany). After adding 300 μl EDTA (0.5 M) and 200 μl 1× TE-buffer, samples were incubated (1 min, RT), centrifuged (2 min, 16000*g*, RT), and the supernatants were carefully removed. Bacterial DNA in the remaining pellets was subsequently isolated following the manufacturer’s instructions of the kit. DNA was eluted in 50 μl elution buffer and stored at − 20 °C until use.

### Reconstruction of *aprX* and *rpoB* single-gene phylogenies

Protein-coding genes of 61 *Pseudomonas* strains were predicted based on NCBI genome assemblies (Supplementary Table S2) using Prodigal v2.6 (Hyatt et al. 2010). *AprX* and *rpoB* gene sequences were extracted from gene predictions by searching for unidirectional best BLASTp v2.2.25+ (Camacho et al. 2009) hits to the NCBI reference sequences with GenBank identifiers AGL85010.1 and KKJ93525.1, respectively. Subsequently, multiple sequence alignments were calculated with ClustalW (Thompson et al. 1994) and used for maximum likelihood phylogenetic tree reconstruction via the MEGAX v10.0.5 software (Kumar et al. 2018) applying the general time reversible (GTR) model under the assumption of rate heterogeneity (+G) and a proportion of invariant sites (+I). To infer branch confidence values, 500 bootstrap replicates were computed for each tree. Finally, both phylogenies were visualized using the interactive Tree Of Life (iTOL) v5.3 online tool (Letunic and Bork 2019). The strain *P. aeruginosa* WS 5022 served as outgroup to root the trees.

### Estimation of *aprX* and *rpoB* sequence similarities

Pairwise p-distances of *aprX* and *rpoB* gene sequences from *Pseudomonas* strains were calculated based on single-gene multiple sequence alignments using the MEGAX v10.0.5 software (Kumar et al. 2018). After subtracting distance values from 1 and multiplication by a factor of 100, pairwise sequence similarities were obtained. The outgroup strain *P. aeruginosa* WS 5022 was excluded from the comparison.

### Primer and probe design

All primer and hydrolysis probes used in this study and their main characteristics are listed in Table 1. In total, five species-specific hydrolysis probes and primers targeting *aprX* were created to detect the following species: *P. proteolytica*, *P. gessardii* and *P. gessardii*-like species (probe 1; P1); *P. fluorescens, P. lactis* and *P. lactis*-like species (probe 2; P2); *P. protegens* and *P. protegens*-like species (probe 3; P3); *P. fragi* (probe 4; P4); *P. lundensis* and *P. lundensis*-like species (probe 5; P5). Additionally, one universal *rpoB* probe (probe 6; P6) and primer pair, targeting all members of *Pseudomonas*, were produced. For design, *aprX* and *rpoB* sequences of 61 *Pseudomonas* strains (30 type strains and 31 environmental isolates) were selected. All isolates and associated genome accession numbers are listed in the Supplementary Table S2. Respective sequences were aligned applying MEGA X (Kumar et al. 2018) and conserved regions were identified manually and checked for suitability. The formation of self- and cross-dimers of primers and probes was analyzed using Multiple Primer Analyzer (Thermo Fisher Scientific Inc., Waltham, Massachusetts), and hairpin formation was tested via OligoCalc (Kibbe 2007). Resulting primers had an annealing temperature between 55.2 and 58.2 °C, a GC content of 47.6 to 64.7%, and a length of 17 to 21 nucleotides. Hydrolysis probes revealed an annealing temperature between 63.1 and 65.8 °C, a GC content of 56.5 to 68.4%, and a length between 19 and 23 nucleotides. All oligonucleotides and hydrolysis probes were obtained from Eurofins Genomics Germany GmbH (Ebersberg, Germany).

**Table 1:** Composition and characteristics of the two triplex qPCR assays. Assay set 1 targets abundant, high proteolytic *Pseudomonas* species, and set 2 detects abundant, but less proteolytic species and total pseudomonads. Sequences of six primer pairs (Pr_F and Pr_R) and hydrolysis probes (P1–P6), final concentrations, amplicon length, target genes, and target species are listed. Probes’ fluorophores (5′-ends) and quenchers (3′-ends) are shown in bold

Primer/probe	Sequence (5′-3′)	Conc. [nM]	Amplicon length [bp]	Target gene	Target species
Assay set 1					
Pr1_ F	GCACCAATGASAAGTACCACA	400	135	*aprX*	*P. proteolytica*, *P. gessardii*, *P. gessardii*-like
Pr1_R	GTATGGCCGATCTCGTGG	600			
P1	**CY5**-CACGGATGGCACCTCGTGGTAC-**BHQ2**	200			
Pr2_F	ACCTTCCTCACCTCGGCT	600	137	*aprX*	*P. fluorescens*, *P. lactis*, *P. lactis*-like
Pr2_R	GGTAAAGGTCACGTTGGCA	600			
P2	**TexasRed**-AACACCCAGCAGAAAGCACAGGC-**BHQ2**	200			
Pr3_F	GCATCTGCCGAACAACAAC	400	85	*aprX*	*P. protegens, P. protegens*-like
Pr3_R	CGATCGTATTGGTGGCTGA	200			
P3	**FAM**-CCGCAGCAAGTTCGGCGTATAAC-**BHQ1**	150			
Assay set 2					
Pr4_F	AGCAGCATTGTCCGTTGG	400	130	*aprX*	*P. fragi*
Pr4_R	CGGTGGTGAGCGAAGGT	600			
P4	**FAM**-CGGCAAACACCGGCAGTTCTG-**BHQ1**	200			
Pr5_F	TGCTGGCCTGGTTGTAGC	600	92	*aprX*	*P. lundensis, P. lundensis*-like
Pr5_R	TCACCGGGATTACTCATCTCA	600			
P5	**TexasRed**-ACGACCGCATCACCCGCCT-**BHQ2**	200			
Pr6_F	CAGCCGYTGGGTGGTAA	400	130	*rpoB*	*Pseudomonas* spp.
Pr6_R	CCGTTCACATCGTCCGA	200			
P6	**CY5**-AGTTCGGTGGTCAGCGTTTCGG-**BHQ2**	150			

### qPCR optimization and conditions

Quantitative PCR was performed with the real-time PCR detection system CFX96/C1000 Touch^TM^ (Bio-Rad Laboratories Inc., Hercules, CA, USA) using the CFX Maestro^TM^ software and the following reaction conditions: Initial denaturation step at 95 °C for 2 min and 35 cycles including denaturation at 95 °C for 5 s and annealing/extension at 61 °C for 15 s. Optimal primer concentration was determined separately for each primer pair via a SYBR green qPCR assay. For this, a total reaction volume of 10 μl was used, including 5 μl SsoAdvanced^TM^ Universal SYBR**®** Green Supermix (Bio-Rad Laboratories Inc., Hercules, CA, USA), 1 μl per primer in different concentrations (200 nM, 400 nM, or 600 nM), and 1 μl target DNA. The optimal quantity of the hydrolysis probes was subsequently determined by applying different probe concentrations (150 nM, 200 nM, 250 nM) with the previously defined primer concentrations in singleplex probe-based qPCR. Singleplex and multiplex probe-based qPCR was performed in 10 μl reaction volume, containing 5 μl of the SsoAdvanced™ Universal Probes Supermix (Bio-Rad Laboratories Inc., Hercules, CA, USA), primers, and probe in optimized concentrations (Table 1) and 1 or 2 μl DNA template, depending on the experiment. Multiplex qPCR utilizing six probes was split into two triplex assays, containing each three hydrolysis probes and the respective primer pairs as listed in Table 1.

### Production of artificial DNA mixtures

In order to evaluate the multiplex qPCR assays, complex DNA pools were generated to be used as templates. Two strains per target species of each species-specific probe (P1–P5) were selected as representatives. Strains were grown on TS-agar plates for 24 h, and DNA was extracted using the QIAamp DNA Mini Kit (Qiagen N.V., Hilden, Germany) according to manufacturer’s specifications. Then, tenfold dilution series containing 100 to 0.01 ng/μl DNA of each strain were prepared and concentrations were checked using the Qubit 2.0 Fluorometer (Invitrogen AG, Carlsbad, CA, USA). Subsequently, identical concentrations of five target DNAs, each of which is detected by one of the five species-specific probes, were combined in a single DNA pool. In total, six distinct DNA mixtures (pool 1–6) with various target DNA compositions were prepared, which are summarized in Supplementary Table S3. In addition, each DNA mixture was generated in five different total DNA concentrations (0.01, 0.1, 1.0, 10, and 100 ng/μl) using the dilution series of the single target DNAs. Thus, the final concentration of target DNAs per species-specific probe (P1–P5) was between 0.002 and 20 ng/μl in the DNA pools. With regard to the universal *rpoB* probe (P6), detecting all pseudomonads, target DNA concentration ranged from 0.01 to 100 ng/μl in the DNA mixtures.

### Amplification efficiency and sensitivity of the qPCR assays

For each hydrolysis probe, singleplex qPCR was performed before conducting multiplex qPCR, in order to check for probe functionality and possible interfering interactions between primer and probes. For both single- and multiplex qPCR, all six DNA mixtures (Supplementary Table S3) were used in different concentrations as templates. The quantification cycle (*C*_q_) values obtained per probe from differing target DNA concentrations in the singleplex approach were compared with corresponding *C*_q_ values received from multiplex qPCR. For the determination of reaction efficiencies, regression lines were created by plotting the *C*_q_ values versus the log of the target DNA concentration used for qPCR. The amplification efficiency (*E*) was calculated for each probe from the slopes using the formula: $E={10}^{\frac{-1}{\mathrm{slope}}}-1$

In order to evaluate the sensitivity of the qPCR assay, the linear dynamic range was determined as well as the lower limit, defined as the number of detectable gene copies when applying the minimum target DNA concentration (0.002 ng for probes P1–P5 and 0.01 ng for probe P6). Copy numbers of *aprX* (for P1–P5) and *rpoB* (P6) were calculated for each probe separately, using the following formula (Staroscik 2011-2020):

$$ \mathrm{gene}\ \mathrm{copy}\ \mathrm{number}=\frac{\mathrm{amount}\ \mathrm{of}\ \mathrm{genomic}\ \mathrm{DNA}\ \left[\mathrm{ng}\right]\times \mathrm{Avogadro}\ \mathrm{constant}\ \left(6.022\times {10}^{23}\left[\frac{1}{\mathrm{mol}}\right]\right)}{\left(\ \mathrm{genome}\ \mathrm{size}\times \mathrm{mass}\ \mathrm{of}\ \mathrm{dsDNA}\ \left(660\ \left[\frac{\mathrm{g}}{\mathrm{mol}}\right]\right)\right)\times {10}^9\frac{\mathrm{ng}}{\mathrm{g}}}. $$

As *aprX* and *rpoB* present single-copy genes, the genome number is equivalent to the number of gene copies. Genome sizes of 18 target strains, which were used for the production of the DNA mixtures, were taken from NCBI and averaged per probe: 6265591 bp (P1), 6528297 bp (P2), 6799673 bp (P3), 5227135 bp (P4), 5131361 bp (P5), and 6171092 bp (P6). The average molecular mass per base pair (dsDNA) was defined as 660 g/mol.

### Specificity of qPCR assays

In addition to 26 *Pseudomonas* strains, belonging to the seven target species (plus five very closely related species), 49 *Pseudomonas* strains from 45 non-target species and isolates of 40 other bacterial species were selected in order to check the specificity of the qPCR assays (Supplementary Table S1). Selection of strains was based on their relevance in raw milk and their phylogenetic proximity to the target species. Strains were grown on TS-agar plates for 24 h, and DNA was extracted using the QIAamp DNA Mini Kit (Qiagen N.V., Hilden, Germany) according to manufacturer’s specifications. DNA concentration was then measured via Qubit 2.0 Fluorometer (Invitrogen AG, Carlsbad, CA, USA) and adjusted to 1–2 ng/μl. One microliter DNA was used as template for the two triplex qPCR assays in a 10 μl reaction volume leading to a final DNA concentration of 0.1–0.2 ng/μl.

### Generation of standard curves in raw milk

For the correlation of *C*_q_ values and cell counts, three to nine target strains were selected per species-specific probe and all of them (in total 26 strains) for the universal probe (Supplementary Table S1 in bold). Strains were grown in TSB at 30 °C and 150 rpm for 16 h, before 2 ml of each overnight culture was centrifuged (1 min, 13000*g*, RT). The supernatant was discarded, and the pellet resuspended in 10 ml of fresh raw milk. Afterward, a fivefold dilution series (1:5^1^ to 1:5^9^) of this sample was prepared with fresh raw milk. The dilution steps 1:5^2^, 1:5^3^, 1:5^5^, 1:5^7^, and 1:5^9^ were selected for cell count determination by plating and DNA extraction using the DNeasy® PowerFood® Microbial Kit (Qiagen N.V., Hilden, Germany) with the above-listed protocol. Extracted, bacterial DNA of each strain was then applied as template (2 μl in 10 μl reaction volume) in the two triplex qPCR assays, and *C*_q_ values were determined of all samples in two technical replicates. For the *rpoB* probe (P6), the dilution step 1:5^2^ was not considered due to the high amount of target DNA. In parallel, *Pseudomonas* counts of all sample dilutions were quantified on CFC-agar plates. The *Pseudomonas* count of untreated raw milk was determined (1.6 × 10^3^ cfu/ml) and subtracted from the counts of spiked raw milk samples. For standard curves, logarithmic cell counts per milliliter were plotted against the respective *C*_q_ values of identical samples.

## Results

### Setup of multiplex qPCR for species-specific and total *Pseudomonas* detection

Besides the quantification of total pseudomonads, the novel qPCR assay was developed to specifically detect seven milk-relevant *Pseudomonas* species, namely *P. proteolytica*, *P. gessardii*, *P. lactis*, *P. fluorescens*, *P. protegens*, *P. lundensis*, and *P. fragi.* As target genes, *aprX* was chosen for species-specific detection, and the conserved *rpoB* gene for the quantification of total *Pseudomonas* counts.

**Fig. 1:**
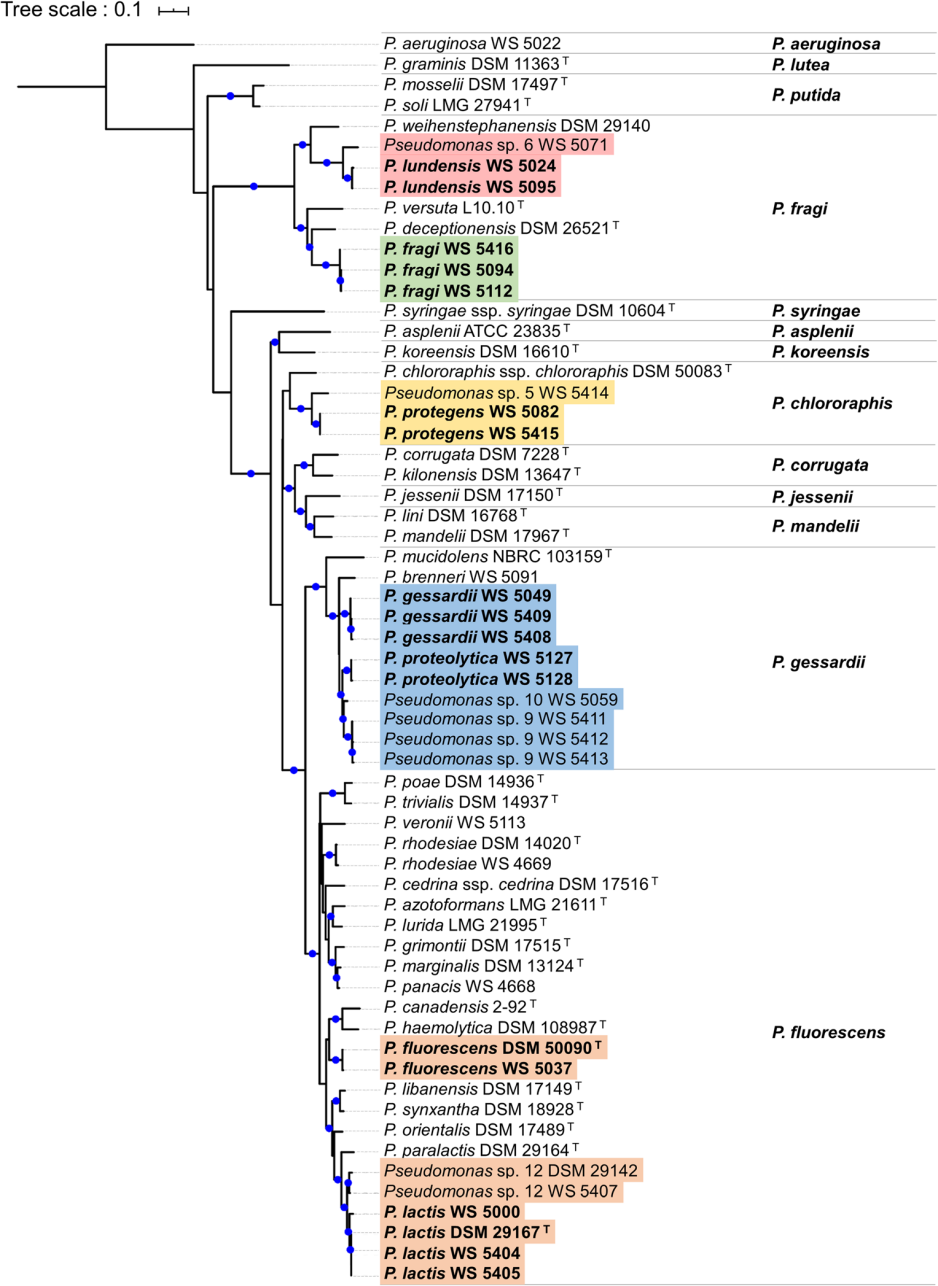
Maximum likelihood phylogeny of the *aprX* gene based on 1,296 positions in the multiple sequence alignment of 61 distinct *Pseudomonas* strains. Molecular evolution was inferred by the GTR+G+I model, and the tree was outgroup-rooted (*P. aeruginosa* WS 5022). Branches with high bootstrap support (≥ 70% of 500 replicates) are marked with blue circles. Target strains of the species-specific probes are highlighted in blue (P1), orange (P2), brown (P3), green (P4), and red (P5). Strains were assigned to 13 monophyletic groups, whose names are listed in bold to the right of the tree

In order to check the suitability of the selected target genes and to determine the number of probes and primers needed, phylogenetic analyses using *aprX* and *rpoB* sequences of 61 *Pseudomonas* strains, assigned to 46 different species, were performed. The overall topology of the phylogenetic *aprX* tree was similar to the one based on *rpoB* sequences (Fig. 1 and Supplementary Fig. S1), considering the classification of strains into the previously described *Pseudomonas* subgroups (Gomila et al. 2015; Peix et al. 2018; Maier et al. 2020) and the distribution of species within these groups. However, the *aprX* sequences were more variable and discriminative (66.0–100.0% sequence similarity range), thus enabling a higher resolution than the conserved *rpoB* gene sequences (85.1–100.0% sequence similarity range), which served for the design of a genus-specific probe and respective primers. With respect to the seven chosen target species, the *aprX* tree exhibited a distribution of the 18 representative strains in four *Pseudomonas* subgroups, namely *P. fluorescens*, *P. gessardii*, *P. fragi*, and *P. chlororaphis*. Sequences of isolates from *P. lactis* and *P. fluorescens*, both belonging to the *P. fluorescens* subgroup, as well as the ones from *P. proteolytica* and *P. gessardii*, being part of the *P. gessardii* subgroup, showed a very high inter-species sequence similarity of at least 91.9 and 94.1%, respectively. For these very closely related species, the design of a single hydrolysis probe and primer pair, targeting the *aprX* sequences from members of both species, was possible. In contrast, the *aprX* sequences of *P. lundensis* and *P. fragi* strains, all belonging to the *P. fragi* subgroup, differed largely (maximum inter-species sequence similarity of 81.1%), and therefore, separate probes and primers were created for each species. Also, for *P. protegens* strains, being located in the *P. chlororaphis* subgroup, the design of an additional probe plus primers was necessary. Consequently, a total of five hydrolysis probes (P1–P5) and primer pairs were generated to detect all seven target species. Besides the 18 strains of the target species, eight very closely related isolates were taken into account for probe and primer design (Supplementary Table S2), as they were shown to be frequently present in raw milk and exhibit comparable proteolytic characteristics (von Neubeck et al. 2015; Maier et al. 2020). According to our phylogenomic study, these strains do not belong to species with validly described names (Maier et al. 2020) and will be referred to as “*P. lactis*-like,” “*P. gessardii*-like,” “*P. lundensis*-like,” and “*P. protegens*-like” in the following.

The six hydrolysis probes and respective primer pairs were split into two triplex qPCR reactions, whose compositions are summarized in Table 1. Assay 1 comprised three probes (P1–P3) and primer pairs to quantify common and highly proteolytic species, namely *P. proteolytica*, *P. gessardii* and *P. gessardii-like* species (P1); *P. fluorescens*, *P. lactis *and *P. lactis-*like species (P2); as well as *P. protegens* and *P. protegens*-like species (P3). Assay 2 contained two probes (P4 and P5) and respective primers detecting *P. fragi* (P4), and *P. lundensis* plus *P. lundensis*-like species (P5), which are less proteolytic, but abundant in raw milk samples. Moreover, assay 2 was complemented with the universal *Pseudomonas* primers and probe P6 for quantification of total *Pseudomonas* counts. For all designed primers and probes, the optimal concentrations were specified separately by singleplex qPCR (Table 1), and an optimal annealing temperature of 61 °C was determined by gradient qPCR (data not shown).

### Efficiency and linearity of single- and multiplex qPCR

For determination of the amplification efficiencies, six defined DNA pools of target and non-target DNA were produced (Supplementary Table S3) and various dilutions thereof were applied as templates in qPCR. After testing each probe-primer combination separately in a singleplex assay, three probes plus primers were combined in the triplex approach. Therefore, DNA of two to six different target strains was applied for each of the five species-specific probes (P1–P5), and DNA of all 18 target strains was employed for the universal *Pseudomonas* probe (P6). Averaged *C*_q_ values were calculated for each hydrolysis probe with its primer pair (Table 2). Thereby, linear correlations between *C*_q_ values and DNA concentrations were observed for all six probes in singleplex and multiplex qPCR, yielding high correlation coefficient (*R*^2^) values of > 0.975 and PCR amplification efficiencies (E) of 85–97%. Since mean *C*_q_ values of singleplex and multiplex qPCRs were highly comparable (Table 2), possible interactions between the different probes and primer pairs in the multiplex reactions do not adversely affect target detection or amplification.

**Table 2:** Correlation between *C*_q_ values and DNA concentration using the six *Pseudomonas* probes (P1–P6) in singleplex and multiplex qPCR. As templates, serial dilutions of six artificial DNA pools (1–6) were applied, each containing DNA of five different *Pseudomonas* strains in equal quantities (Supplementary Table S3). In total, DNA of six different target strains (from pool 1 to 6) was measured for the species-specific probes P1 and P2, and DNA of each two strains (from pool 1 and 2) for probes P3, P4, and P5. For the universal *Pseudomonas* probe P6, all 18 strains from the six DNA pools were taken into account. All measurements were conducted in two technical replicates, and averaged *C*_q_ values, amplification efficiency, and coefficients of determination are shown

Probe	Template DNA from	Concentration of target DNA [ng/μl]	Singleplex qPCR		Multiplex qPCR	
			Mean *C*_q_ value	Efficiency /* R*^2^ value	Mean *C*_q_ value	Efficiency /* R*^2^ value
P1	6 target strains (pool 1–6)	2	17.22 ± 0.63	87% 0.979	16.06 ± 0.58	85% 0.981
		0.2	20.44 ± 0.52		19.46 ± 0.40	
		0.02	24.18 ± 0.55		23.25 ± 0.60	
		0.002	28.24 ± 0.60		27.30 ± 0.64	
		0.0002	32.44 ± 1.28		31.65 ± 1.35	
P2	6 target strains (pool 1–6)	2	15.72 ± 0.69	89% 0.990	15.49 ± 0.21	90% 0.992
		0.2	19.50 ± 0.15		19.12 ± 0.51	
		0.02	23.02 ± 0.15		22.57 ± 0.42	
		0.002	26.65 ± 0.36		26.29 ± 0.23	
		0.0002	30.72 ± 0.91		30.25 ± 0.39	
P3	2 target strains (pool 1 + 2)	2	16.77 ± 0.16	90% 0.998	16.04 ± 0.25	88% 0.995
		0.2	20.21 ± 0.24		19.39 ± 0.13	
		0.02	23.92 ± 0.25		22.90 ± 0.24	
		0.002	27.94 ± 0.25		27.03 ± 0.11	
		0.0002	32.37 ± 0.31		31.25 ± 0.07	
P4	2 target strains (pool 1 + 2)	2	16.20 ± 0.06	92% 0.999	14,70 ± 0.20	93% 0.993
		0.2	19.52 ± 0.20		17,88 ± 0.30	
		0.02	23.03 ± 0.07		21,31 ± 0.15	
		0.002	27.01 ± 0.08		25,26 ± 0.08	
		0.0002	31.37 ± 0.55		29,74 ± 0.43	
P5	2 target strains (pool 1 + 2)	2	15.50 ± 0.09	94% 0.998	14.99 ± 0.14	97% 0.998
		0.2	18.96 ± 0.07		18.28 ± 0.12	
		0.02	22.29 ± 0.06		21.58 ± 0.16	
		0.002	26.10 ± 0.19		25.21 ± 0.16	
		0.0002	30.71 ± 0.51		29.80 ± 0.56	
P6	18 target strains (pool 1–6)	10	13.91 ± 0.44	93% 0.989	13.20 ± 0.11	95% 0.998
		1	17.48 ± 0.38		16.43 ± 0.11	
		0.1	20.87 ± 0.44		19.90 ± 0.21	
		0.01	24.41 ± 0.38		23.54 ± 0.14	
		0.001	28.42 ± 0.46		28.2 3 ± 0.51	

For the five species-specific probes (P1–P5), target DNA amounts from 2 to 0.0002 ng/μl (final concentrations in 10 μl reaction volume) and for the universal *Pseudomonas* probe P6 from 10 to 0.001 ng/μl were detectable, demonstrating a wide linear dynamic range over 4 log-steps. For qPCR with P1–P5, calculated minimal *aprX* gene copy numbers lay between 268 and 356. Using the universal *Pseudomonas* probe (P6), a minimum of approx. 1.5 × 10^3^ *rpoB* gene copies was detectable with 0.001 ng/μl target DNA. Since no greater *C*_q_ value than 32.5 was received for all probes when applying the lowest target DNA amounts, a cut-off value of 33 was defined for further experiments, and higher *C*_q_ values were considered unquantifiable.

### Specificity of the multiplex qPCR assay

To verify the specificity of the assay, 1–2 ng/μl genomic DNA of 75 *Pseudomonas* strains (target and non-target strains), assigned to 57 different species, and of 40 other bacterial species was applied as template in the qPCR assays. For strain selection, *Pseudomonas* isolates of the target species and their closest relatives, as well as representatives of the whole genus, were considered. Other bacterial species were chosen due to their phylogenetic proximity to the genus *Pseudomonas* and/or their relevance in milk and milk products.

Using the five species-specific probes (P1–P5) targeting *aprX*, all 18 strains of the defined seven target species were detected successfully, yielding *C*_q_ values from 18.85 to 22.13 (Table 3). Moreover, the eight very closely related isolates, which belong to *P. gessardii*-like, *P. lactis*-like, *P. lundensis*-like, and *P. protegens*-like species, resulted in positive signals in the same range (*C*_q_ 19.59–21.27).

**Table 3:** *C*_q_ values from triplex qPCR assays, applying five species-specific probes (P1–P5) and the universal *Pseudomonas* probe (P6). Genomic DNA from 26 target strains was used as template in a final concentration of 0.1–0.2 ng/μl. Mean values of two technical replicates per measurement are shown. Hyphens (-) represent no signal in qPCR. *C*_q_ values above the defined threshold of 33 were considered as not quantifiable and are given in brackets

	Species-specific hydrolysis probes					Universal *Pseudomonas probe*
Target Strains (P1–P5)	P1	P2	P3	P4	P5	P6
*Pseudomonas gessardii* WS 5049^P1^	21.05	-	-	-	-	20.70
*Pseudomonas gessardii* WS 5408^P1^	21.05	-	-	-	-	19.52
*Pseudomonas gessardii* WS 5409^P1^	21.02	-	-	-	-	19.73
*Pseudomonas proteolytica* WS 5127^P1^	20.08	-	-	-	-	20.51
*Pseudomonas proteolytica* WS 5128^P1^	19.68	-	-	-	-	20.16
*Pseudomonas* sp. 10 WS 5059^P1^	19.59	-	-	-	-	19.89
*Pseudomonas* sp. 9 WS 5411^P1^	20.46	-	-	-	-	20.41
*Pseudomonas* sp. 9 WS 5412^P1^	20.86	-	-	-	-	19.95
*Pseudomonas* sp. 9 WS 5413^P1^	20.67	-	-	(34.81)	-	20.51
*Pseudomonas fluorescens* DSM 50090^T,P2^	-	22.13	-	-	-	20.83
*Pseudomonas fluorescens* WS 5037^P2^	-	20.32	-	-	-	20.00
*Pseudomonas lactis* DSM 29167^T,P2^	-	20.64	-	-	-	19.48
*Pseudomonas lactis* WS 5000^P2^	-	20.03	-	-	-	19.06
*Pseudomonas lactis* WS 5404^P2^	-	21.86	-	-	-	20.39
*Pseudomonas lactis* WS 5405^P2^	-	21.72	-	-	-	19.73
*Pseudomonas* sp. 12 DSM 29142^P2^	-	21.05	-	-	-	20.61
*Pseudomonas* sp. 12 WS 5407^P2^	-	21.27	-	-	-	20.53
*Pseudomonas protegens* WS 5082^P3^	-	-	21.59	-	-	20.25
*Pseudomonas protegens* WS 5415^P3^	-	-	20.56	-	-	19.14
*Pseudomonas* sp. 5 WS 5414^P3^	-	-	20.48	-	-	19.12
*Pseudomonas fragi* WS 5094^P4^	(33.26)	-	-	19.35	-	19.00
*Pseudomonas fragi* WS 5112^P4^	(33.46)	-	-	18.85	-	18.53
*Pseudomonas fragi* WS 5416^P4^	-	-	-	-	-	19.13
*Pseudomonas lundensis* WS 5024^P5^	-	-	-	-	18.57	19.00
*Pseudomonas lundensis* WS 5095^P5^	-	-	-	-	18.86	19.29
*Pseudomonas* sp. 6 WS 5071^P5^	(33.47)	-	-	-	20.00	18.44

^P1,P2,P3,P4,P5^: target strain of probe P1–P5. ^T^: type strain

For the 50 non-target pseudomonads tested, no false-positive signals were received using P3, P4, and P5, demonstrating a very high specificity. However, P1 and P2, detecting multiple target species at once, showed few false-positive results (Supplementary Table S4). In case of P1, a very weak signal was measured with DNA of *P. marginalis* DSM 17967 (*C*_q_ 32.1). Regarding P2, detecting *P. lactis* and *P. fluorescens*, false-positive signals were obtained for four strains of the closely related species *Pseudomonas haemolytica*, *Pseudomonas paralactis*, *Pseudomonas orientalis*, and *Pseudomonas synxantha* (*C*_q_ 24.4–27.6). For the 40 other bacterial species tested, no cross-reactivity was observed using P1–P5, except a negligible signal for *Streptococcus pyogenes* DSM 2071 (*C*_q_ 32.91) (Supplementary Table S4), underlining the high specificity of the designed primers and probes.

Via the universal *rpoB* probe (P6), 74 out of 75 *Pseudomonas* strains tested were successfully detected, generating *C*_q_ values between 17.45 and 22.69 (Supplementary Table 3 and Supplementary Table S4). Only the signal received from DNA of *Pseudomonas stutzeri* WS 5018 was considerably weaker (*C*_q_ 27.13). When testing the 40 other bacterial species, very weak unspecific signals were obtained for 5 isolates with P6 (Supplementary Table S4). Among them, *Pseudoalteromonas haloplanktis* WS 5482 yielded the highest signal with a *C*_q_ of 29.61, while the others showed even higher *C*_q_ values ranging from 30.96 to 32.91.

### Quantification of *Pseudomonas* via multiplex qPCR

In order to generate standard curves for quantification of cells, *C*_q_ values from multiplex qPCR were correlated with the corresponding *Pseudomonas* counts from cultivation. Therefore, 26 *Pseudomonas* target strains were chosen (Supplementary Table S1), and cells were serially diluted in fresh raw milk. From selected dilution steps, cell counts were determined by cultivation, and in parallel, DNA was extracted and used as template for the two triplex qPCR assays.

**Fig. 2:**
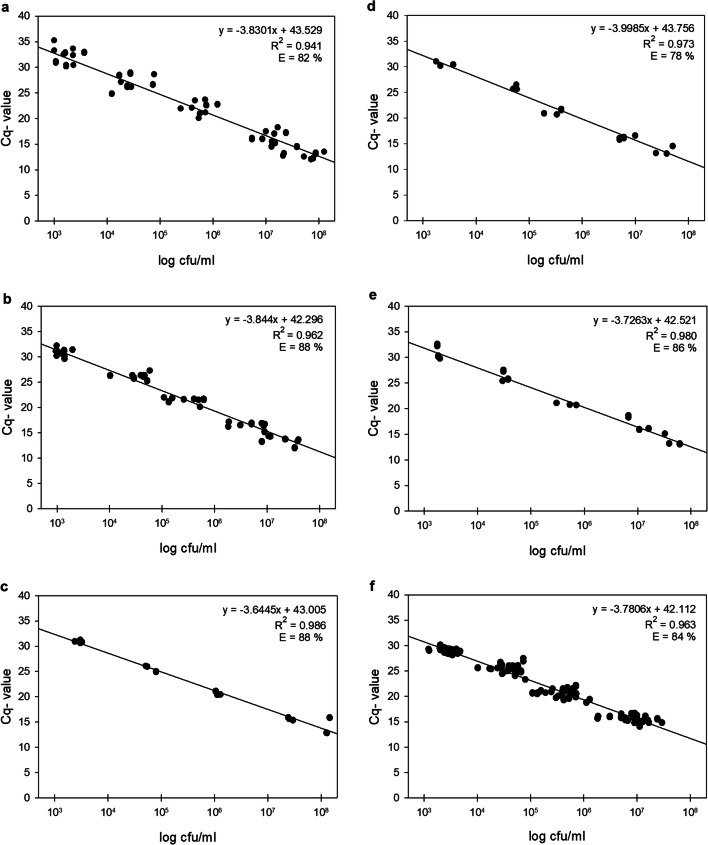
Standard curves of *C*_q_ values from multiplex qPCR using the six *Pseudomonas* probes P1–P6 (**a**–**f**), correlated with cell counts of artificially spiked raw milk samples. 26 representative target strains (9 strains for P1; 8 for P2; 3 for P3, P4 and P5; 26 for P6) were chosen, and cells were serially diluted in fresh raw milk (1:5^2^–1:5^9^). Cell counts of diluted samples were determined by cultivation, and in parallel, DNA of the milk samples was isolated and applied as templates in the two triplex qPCR assays. Averaged *C*_q_ values from two technical replicates of each strain were plotted against the respective *Pseudomonas* counts given in log cfu/ml. Regression equation, coefficient of determination (*R*^2^), and amplification efficiency (E) are given for each standard curve

Linear correlation between *Pseudomonas* cell counts and *C*_q_ values was obtained for each of the six hydrolysis probes, revealing *C*_q_ values from 11.9 to 33.0 (Fig. 2). In agreement with the previously defined cut-off value, *C*_q_ values exceeding 33 were not detected. All standard curves yielded good efficiencies of 78–88% and high *R*^2^ values of 0.944 to 0.986. However, the *R*^2^ values of the probes P1, P2, and P6, detecting multiple species, were slightly lower than the ones from P3, P4, and P5, each targeting only a single species (Fig. 2, Table 3). The dynamic range of detection was found to be linear between ~ 10^3^ and 10^7^ cfu/ml for all probes, covering common *Pseudomonas* cell counts in raw milk. The lowest quantifiable cell amounts were also theoretically calculated for the stated *C*_q_ threshold of 33 via the standard curves, resulting in cell counts between 2 and 9 × 10^2^ cfu/ml (P1: 561 cfu/ml; P2: 262 cfu/ml; P3: 556 cfu/ml; P4: 490 cfu/ml; P5: 359 cfu/ml; P6: 869 cfu/ml).

### Application of the qPCR assay to industrial raw milk samples

For assay validation, 60 independent raw milk samples from 13 different German dairies were analyzed. Determination of total and *Pseudomonas* cell counts by cultivation on TSA and CFC-agar, respectively, revealed great differences regarding the relative *Pseudomonas* amounts of samples (Supplementary Table S5).

**Fig. 3:**
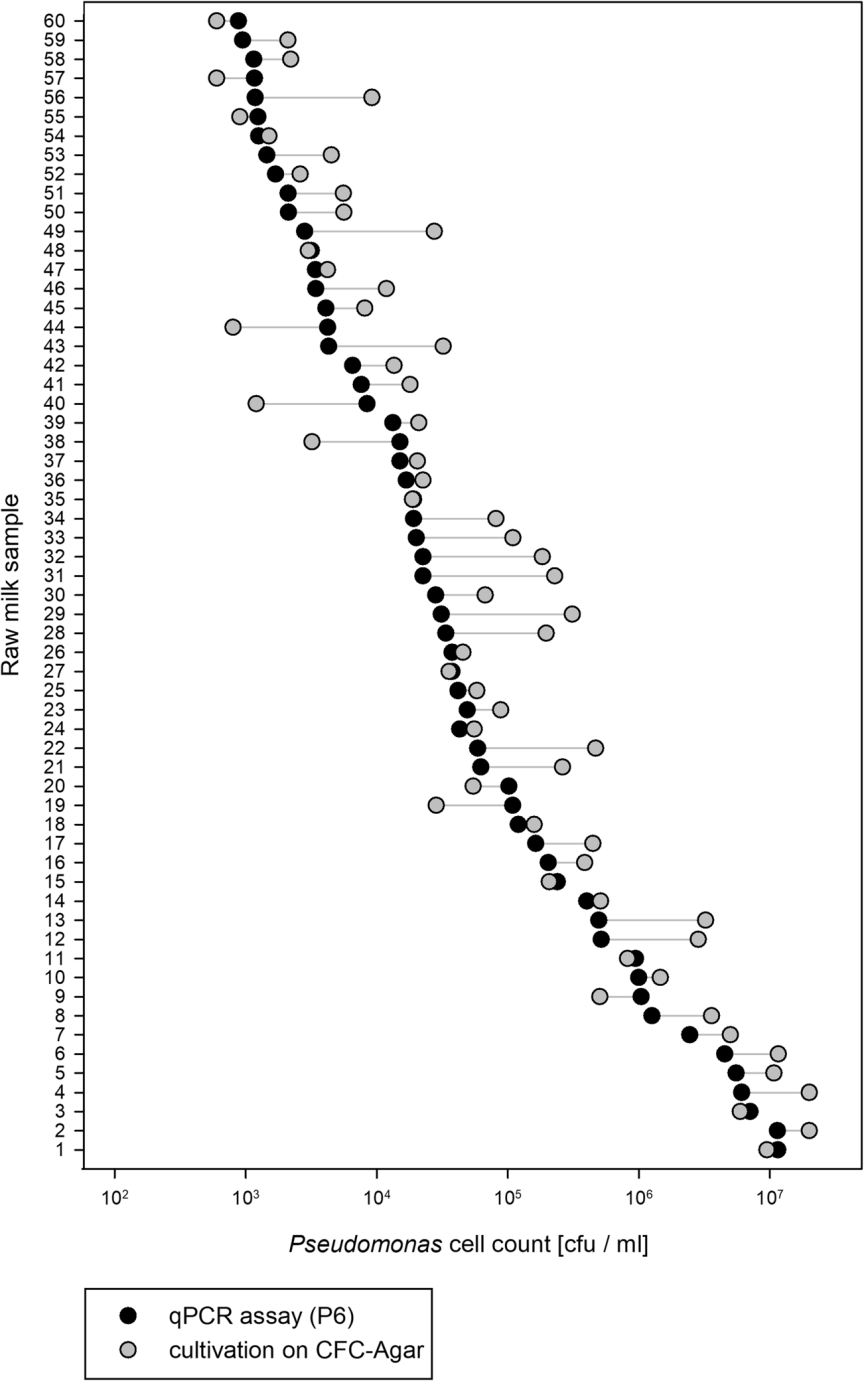
Comparison of *Pseudomonas* cell counts in 60 raw milk samples, determined by multiplex qPCR assay (black dots) and cultivation on CFC-agar (grey dots). For qPCR, DNA was isolated from each raw milk and applied as template in the assay using the universal *Pseudomonas* probe P6. Cell counts were calculated from received *C*_q_ values by linear regression analysis of a standard curve. Deviations between cell counts obtained with multiplex qPCR assay and plating are indicated by grey lines

For qPCR, bacterial DNA was isolated from raw milk samples and used as template in the two triplex assays. Based on the *C*_q_ values obtained, corresponding cell counts were calculated using the respective standard curve. Total *Pseudomonas* cell counts determined by qPCR via the universal *Pseudomonas* probe P6 ranged from 8.8 × 10^2^ to 1.2 × 10^7^ cfu/ml (Fig. 3). In two-thirds of the milk samples, cell counts from qPCR and from the cultivation approach did not differ more than 0.5 log, demonstrating a high concordance of the results. Almost one-third of samples showed a difference in cell amounts between 0.5 and 1 log, and only 2 from the 60 samples (no. 29 and 31) varied slightly more than 1 log (Fig. 3).

**Fig. 4:**
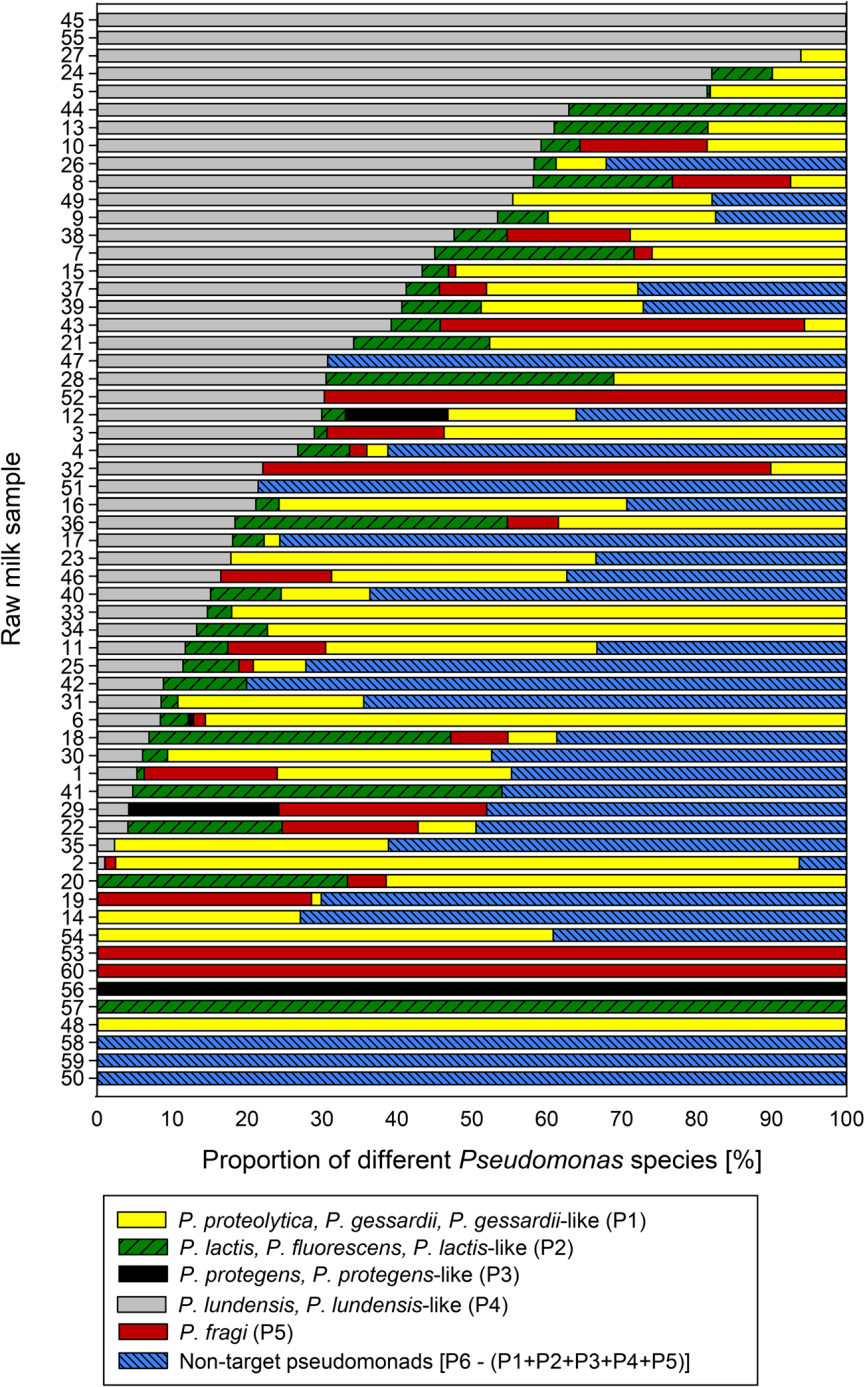
Distribution of different target species and non-target pseudomonads in 60 raw milk samples, determined by qPCR using five species-specific probes (P1–P5) and one universal *Pseudomonas* probe (P6). Cell counts of the target species and total *Pseudomonas* counts were calculated for each sample via standard curves and *C*_q_ values of the respective probes. Results from P6, representing the total count, were defined as 100%, and proportions of target strains accordingly determined. The proportion of non-target pseudomonads was defined by subtracting the sum of all target species (P1–P5) from the total *Pseudomonas* counts (P6)

Regarding the species composition, target species of the five species-specific probes (P1–P5) were detected by qPCRs in all but three raw milk samples tested, while non-target pseudomonads were detected in 53% of the samples (Fig. 4). Remarkably, the occurrence and proportion of each target species differed strongly among the raw milk samples. For each of the five species-specific probes, at least one milk sample contained exclusively the respective target species, confirming the usefulness of the chosen targets (Fig. 4). In terms of frequency and distribution, the species *P. lundensis* and/or *P. lundensis*-like (targeted by P5) were the most common species, being present in 80% of the milk samples and constituting the largest proportion of the *Pseudomonas* population in 27% of the milk samples. The target species of P1, namely *P. proteolytica*, *P. gessardii* and/or *P. gessardii*-like, were similarly frequent, being identified in 73% of the samples and predominant in 23% of the samples. The target species of P2 (*P. fluorescens*, *P. lactis* and/or *P. lactis*-like), P4 (*P. fragi*), and P3 (*P. protegens* and/or *P. protegens*-like) were detected in 60%, 42%, and 7% of the milk samples and accounted for the major share of pseudomonads in only 7%, 8%, and 2% of the samples, respectively. Finally, non-target pseudomonads presented the largest proportion in 33% of the raw milk samples (Fig. 4).

In general, no correlation between total *Pseudomonas* cell counts and distribution of certain target species was observed in the analyzed raw milk samples; however, samples with *Pseudomonas* counts < 10^4^ cfu/ml tended to comprise more different species than samples with higher cell counts.

## Discussion

For the food industry, sensitive and rapid detection methods are crucial to perform a risk assessment and ensure the safety and quality of its products. In recent years, the development of multiplex qPCR assays to detect specific microorganisms in various food matrices has increased rapidly. Utilizing several probes with diverse fluorophores attached, multiplex qPCR enables the co-amplification and differentiation of multiple targets in a single reaction, presenting a cost- and time-saving alternative to singleplex qPCR or cultivation-dependent methods. So far, the majority of these applications allow the identification of foodborne pathogens. For example, multiplex qPCR assays have been developed for the detection of *Salmonella* spp., *Bacillus cereus*, *Listeria monocytogenes*, *Staphylococcus aureus*, *Escherichia coli*, *Campylobacter* spp., or *Vibrio* spp. in various foods (Hong et al. 2007; Tebbs et al. 2011; Forghani et al. 2016; Liu et al. 2017; Heymans et al. 2018; Parichehr et al. 2019). In addition, several applications deal with probiotic or beneficial organisms, such as yeasts, *Acetobacter* spp., or different lactic acid bacteria (LAB) in kefir or starter cultures in cheese production (Bottari et al. 2013; Nejati et al. 2020). Moreover, the detection of food-spoiling bacteria, e.g., *Clostridium* spp. in milk and meats or *Bacillus* spp. and *Paenibacillus* spp. in potato salad and milk, has been carried out by multiplex qPCR assays in previous studies (Morandi et al. 2015; Dorn-In et al. 2018; Nakanojp 2020).

For pseudomonads, two non-quantitative multiplex PCR approaches have been performed for different *Pseudomonas* species in meat (Ercolini et al. 2007) and of *P. fluorescens* strains with a biofilm-forming ability (Xu et al. 2017). However, until now, no qPCR assay has been developed for the simultaneous quantification of various milk-spoiling *Pseudomonas* species in raw milk, which would be very useful for quality assessment in the milk industry. The two triplex assays of this study resulted in the successful enumeration of total *Pseudomonas* counts as well as seven prevalent *Pseudomonas* species in raw milk, enabling discrimination of high and low peptidase producers. Regarding sensitivity, the assays exhibited a linear detection range of approx. 10^3^–10^7^ cfu/ml with lowest quantifiable cell numbers of 2 × 10^2^–2 × 10^3^ cfu/ml, depending on the TaqMan probe. These results are similar to the detection or quantification limits of other developed qPCR assays enumerating bacteria in spiked milk samples, e.g., *Paenibacillus* spp. and *Bacillus* spp. (Nakanojp 2020); *E. coli* and *Salmonella* spp. (Zhou et al. 2017); and *S. aureus*, *L. monocytogenes*, and *Salmonella* spp. (Ding et al. 2017). For some qPCR assays identifying foodborne pathogens in dairy products, lower detection limits of < 10^2^ cfu/ml are required and obtained mostly via time-consuming enrichment steps or other sample pre-treatments (Forghani et al. 2016; Heymans et al. 2018; Parichehr et al. 2019). However, as previous studies revealed that average *Pseudomonas* cell counts in raw milk range from 10^2^ to 10^5^ cfu/ml (Leriche and Fayolle 2012; von Neubeck et al. 2015; Skeie et al. 2019), a higher sensitivity regarding the detection limit of our assay is neither necessary nor beneficial for its application.

When tested using 115 target- and non-target strains, our qPCR assay showed a high level of specificity. Only few false-positive signals were obtained for two out of the five species-specific hydrolysis probes, namely for the multi-target P1 and P2. For P2, this can be explained due to the close phylogenetic proximity of the target species *P. lactis* and *P. fluorescens* to the isolates causing false-positive signals (*P. haemolytica* DSM 108987, *P. paralactis* DSM 29164, *P. orientalis* DSM 17489, and *P. synxantha* DSM 18928). As all these strains were shown to be proteolytic, though less abundant in raw milk (von Neubeck et al. 2015; Maier et al. 2020), the signals are negligible or may even contribute to the detection of proteolytic pseudomonads. Moreover, the universal *Pseudomonas* probe (P6) was shown to be highly specific, detecting all 75 tested *Pseudomonas* strains. Among them, all tested isolates from the 15 species that were previously defined as milk relevant were found (von Neubeck et al. 2015; Caldera et al. 2016; Maier et al. 2020). When 40 non-pseudomonads were tested, the universal probe resulted in five very weak false-positive signals, the highest from DNA of *P. haloplanktis* WS 5482 (C_q_ 29.6). This psychrophilic marine bacterium has occasionally been isolated from cheese rind, but plays no role in the microbiota of raw milk (Feurer et al. 2004; Quigley et al. 2011; Almeida et al. 2014).

With respect to the enumeration of total *Pseudomonas* counts using P6, the results were in good agreement with cell counts received from cultivation. For the majority of samples, *Pseudomonas* cell counts quantified on selective agar were slightly higher than calculated cell counts via qPCR. This could be due to the growth of some members of *Enterobacteriaceae* or *Acinetobacter* on CFC-agar (Flint and Hartley 1996; Salvat et al. 1997), which are known to be frequently present in raw milk (Hantsis-Zacharov and Halpern 2007; Baur et al. 2015; von Neubeck et al. 2015; Li et al. 2018; Ribeiro Junior et al. 2018; Breitenwieser et al. 2020). Therefore, the determination of total *Pseudomonas* by our qPCR assay presents a highly specific and faster (3 h versus 2–3 days) alternative to the quantification of total counts by cultivation.

Remarkably, when 60 independent raw milk samples from 13 different dairies were analyzed for assay validation, unique compositions of the seven target species and non-target pseudomonads were detected for all samples. Thereby, *P. lundensis* and *P. lundensis*-like species (P5) were found most frequently (in 80% of the samples), closely followed by *P. proteolytica*, *P. gessardii* and *P. gessardii*-like species (P1). Members of *P. lactis* and *P. fluorescens* (P2) and *P. fragi* (P4) were also rather common (present in 60% and 42% of samples, respectively), while strains of *P. protegens* (P3) were relatively rare. Previous studies identifying the *Pseudomonas* population of raw milk or dairy products revealed the same predominant species, namely *P. lundensis*, *P. proteolytica*, *P. gessardii*, *P. fragi*, and *P. fluorescens*. In contrast, representatives of *P. protegens* were less common (Marchand et al. 2009a; De Jonghe et al. 2011; von Neubeck et al. 2015; Caldera et al. 2016). Besides, in all of these studies, other isolates from partly unclassified *Pseudomonas* species were identified, which is also consistent with our results revealing the presence of non-target species in about half of the samples tested.

Since it was shown that the composition of the *Pseudomonas* population varies greatly in raw milk samples, the proportions of highly, middle, and low proteolytic isolates are strongly different, too. Here, our two triplex qPCR assays offer a very useful tool to quantify and simultaneously distinguish between the most common raw milk species, possessing different proteolytic potentials. As triplex assay 1 detects specifically highly proteolytic *Pseudomonas* species (e.g., *P. proteolytica*, *P. gessardii*, or *P. lactis*) and assay 2 species with weaker peptidase activities (e.g., *P. fragi* and *P. lundensis*), they are well suited to estimate the spoilage potential of raw milk. However, for a more accurate risk assessment, future work is needed in order to determine the exact peptidase concentrations causing negative effects in milk. A previous study revealed product defects of UHT milk that was produced from raw milk contaminated with different *Pseudomonas* species, at peptidase activities of ≥ 0.03 pkat/ml (Stoeckel et al. 2016a). Correlations between AprX amounts and the required cell numbers of high as well as of low proteolytic *Pseudomonas* species are necessary for an informed definition of threshold CFU values, which indicate the probability of product spoilage.

In summary, the novel multiplex qPCR assay provides an accurate and rapid technique to quantify the total *Pseudomonas* counts in raw milk and to distinguish between the most prevalent *Pseudomonas* species with different proteolytic potentials. Thereby, it presents a powerful tool for the dairy industry to predict the spoilage risk and shelf life of raw milk samples at an early stage in order to decide on further processing, e.g., towards UHT or fresh milk products.

## Supplementary Information

ESM 1 (PDF 1.15 mb)

## Data Availability

All data generated during this study are included in the paper or in the electronic supplementary material. Strains and additional raw data are available from the authors upon request.
