# Supplementary material for: Simultaneous quantification of the most common and proteolytic Pseudomonas species in raw milk by multiplex qPCR
Source: Appl Microbiol Biotechnol. 2021 Feb 1;105(4):1693–708. doi: 10.1007/s00253-021-11109-0 (PMC7880948; doi:10.1007/s00253-021-11109-0)
Supplement: Supplementary file 1 — (PDF 1.15 mb) [file 253_2021_11109_MOESM1_ESM.pdf]

Journal: Applied Microbiology and Biotechnology

Title: Simultaneous quantification of the most common and proteolytic *Pseudomonas* species in raw milk by multiplex qPCR

Authors: Christopher Maier[1], Katharina Hofmann[1] Christopher Huptas[2], Siegfried Scherer[1,2], Mareike Wenning[3], Genia Lücking[1*]

[1] ZIEL Institute for Food and Health, Wissenschaftszentrum Weihenstephan, Technische Universität München, Weihenstephaner Berg 1, 85354 Freising, Germany
[2] Lehrstuhl für Mikrobielle Ökologie, Wissenschaftszentrum Weihenstephan, Technische Universität München, Weihenstephaner Berg 3, 85354 Freising, Germany
[3] Bavarian Health and Food Safety Authority (LGL), Veterinärstr. 2, 85764 Oberschleißheim, Germany
[*]corresponding author

Corresponding author: Genia Lücking
Email: genia.luecking@tum.de
Tel; 08161 71 2257

Supplementary Figure

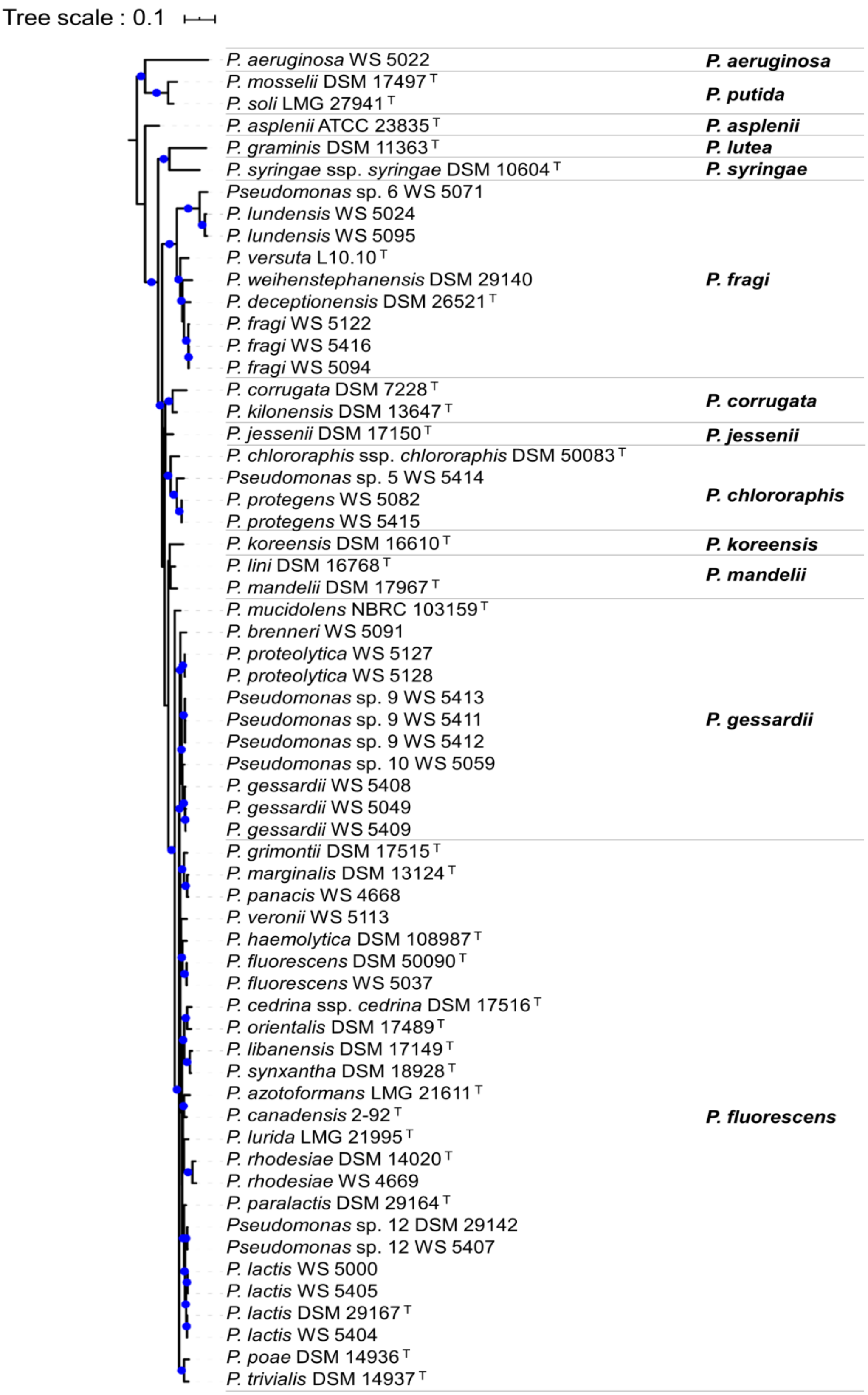

Fig S1 Rooted *rpoB* gene phylogeny comprising 61 different *Pseudomonas* strains. The tree was reconstructed on the basis of 4,074 alignment positions using the maximum likelihood method and the GTR+G+I nucleotide substitution model. *P. aeruginosa* WS 5022 was used as outgroup. Blue circles indicate branches with high bootstrap support ($\geq$ 70 % of 500 replicates). The phylogeny was separated in 13 monophyletic groups. Group names are shown in bold next to the tree

Supplementary Tables

Table S1 Bacterial strains used for evaluation of the multiplex qPCR assay. Target strains of the five species-specific hydrolysis probes (P1, P2, P3, P4 and P5) are indicated in bold print

Species	Strain	Strain collection	Origin
Acinetobacter guillouiae	WS 5457	WS (TUM)	raw milk, Germany
Acinetobacter johnsonii	WS 5458	WS (TUM)	raw milk, Germany
Acinetobacter junii	WS 5459	WS (TUM)	environmental monitoring for pharmaceutical industry
Aerococcus viridans	WS 5460	WS (TUM)	raw milk, Germany
Arthrobacter casei	WS 5462	WS (TUM)	Appenzeller cheese, Germany
Arthrobacter ilicis	WS 5461	WS (TUM)	environmental monitoring for pharmaceutical industry
Bacillus licheniformis	DSM 8785	DSMZ	miilk, unkown
Bifidobacterium longum T	DSM 20219	DSMZ	intestine of adult, unkown
Brevibacterium aurantiacum	WS 5463	WS (TUM)	raw milk, Germany
Brevibacterium pityocampae	WS 5464	WS (TUM)	raw milk, Germany
Chryseobacterium haifense	WS 5465	WS (TUM)	raw milk, Germany
Citrobacter freundii	WS 5466	WS (TUM)	dairy product, Germany
Corynebacterium comporealensis	WS 5467	WS (TUM)	raw milk, Germany
Corynebacterium stationis	WS 5469	WS (TUM)	raw milk, Germany
Delftia lacustris	WS 5470	WS (TUM)	raw milk, Germany
Enhydrobacter aerosaccus	WS 5471	WS (TUM)	environmental monitoring for pharmaceutical industry
Enterobacter cowanii	WS 5472	WS (TUM)	raw milk, Germany
Enterococcus durans	WS 5473	WS (TUM)	raw milk, Germany
Enterococcus italicus	WS 5474	WS (TUM)	raw milk, Germany
Hafnia alvei	DSM 30098	DSMZ	raw milk, unkown
Kocuria kristinae	WS 5475	WS (TUM)	raw milk, Germany
Lactobacillus paracasei	WS 5476	WS (TUM)	Bauernkäse, Germany
Lactococcus garvieae	WS 5478	WS (TUM)	raw milk, Germany
Lactococcus lactis	WS 5479	WS (TUM)	raw milk, Germany
Lactococcus raffinolactis T	DSM 20443	DSMZ	raw milk, unkown
Leuconostoc lactis	WS 5480	WS (TUM)	Unknown
Microbacterium hominis	WS 5481	WS (TUM)	raw milk, Germany
Pseudoalteromonas haloplanktis	WS 5482	WS (TUM)	dairy product, Germany
Pseudomonas aeruginosa	WS 5022	WS (TUM)	raw milk, Germany
Pseudomonas alcaligenes T	WS 5013	DSMZ	dairy product, Germany
Pseudomonas asplenii T	DSM 17133	DSMZ	bird's nest fern *Asplenium nidus,* USA
Pseudomonas brenneri	WS 5091	DSMZ	raw milk, Germany
Pseudomonas canadensis T	LMG 28499	LMG	Soil, Woodslee, Ontario Canada
Pseudomonas cedrina T	DSM 17516	DSMZ	spring water, Lebanon
Pseudomonas chengduensis T	DSM 26382	DSMZ	waste disposal site, China
Pseudomonas chlororaphis T	DSM 50083	DSMZ	plate contaminant, unkown
Pseudomonas corrugata T	DSM 7228	DSMZ	*Lycopersicon esculentum,* UK
Pseudomonas deceptionensis T	DSM 26521	DSMZ	marine sediment, Antarctica

Species	Strain	Strain collection	Origin
Pseudomonas endophytica	WS 5129	WS (TUM)	stem tissue of *Solanum tuberosum*, Spain
Pseudomonas extremaustralis T	DSM 17835	DSMZ	water pond, Antarctica
Pseudomonas fluorescens P2	**WS 5037**	WS (TUM)	raw milk, Germany
Pseudomonas fluorescens T, P2	**DSM 50090**	WS (TUM)	Pre-filter tanks, UK
Pseudomonas fragi P4	**WS 5094**	WS (TUM)	raw milk, Germany
Pseudomonas fragi P4	**WS 5112**	WS (TUM)	raw milk, Germany
Pseudomonas fragi P4	**WS 5416**	WS (TUM)	raw milk, Germany
Pseudomonas gessardii P1	**WS 5049**	WS (TUM)	raw milk, Germany
Pseudomonas gessardii P1	**WS 5408**	WS (TUM)	raw milk, Germany
Pseudomonas gessardii P1	**WS 5409**	WS (TUM)	hok 070719-16-1
Pseudomonas graminis	DSM 100941	DSMZ	cleanroom facility, France
Pseudomonas grimontii T	DSM 17515	DSMZ	mineral water, France
Pseudomonas haemolytica T	DSM 108987	DSMZ	raw milk, Germany
Pseudomonas helleri T	DSM 29165	DSMZ	raw milk, Germany
Pseudomonas jessenii	DSM 10315	DSMZ	soil, Germany, Konstanz
Pseudomonas jessenii	WS 5418	WS (TUM)	raw milk, Germany
Pseudomonas kilonensis T	DSM 13647	DSMZ	agricultural soil, Germany
Pseudomonas koreensis T	DSM 16610	DSMZ	agricultural soil, Korea
Pseudomonas lactis P2	**WS 5404**	WS (TUM)	raw milk, Germany
Pseudomonas lactis P2	**WS 5405**	WS (TUM)	raw milk, Germany
Pseudomonas lactis P2	**WS 5000**	WS (TUM)	raw milk, Germany
Pseudomonas lactis T, P2	**DSM 29167**	DSMZ	raw milk, Germany
Pseudomonas libanensis T	DSM 17149	DSMZ	spring water, Lebanon
Pseudomonas lini T	DSM 16768	DSMZ	rhizospheric soil, France
Pseudomonas lundensis P5	**WS 5024**	WS (TUM)	raw milk, Germany
Pseudomonas lundensis P5	**WS 5095**	WS (TUM)	raw milk, Germany
Pseudomonas mandelii T	DSM 17967	DSMZ	mineral water, France
Pseudomonas marginalis	DSM 13124	DSMZ	*Cichorium intybus,* USA
Pseudomonas mosselii T	DSM 17497	DSMZ	medical specimen, France
Pseudomonas mucidolens	WS 5456	WS (TUM)	raw milk, Germany
Pseudomonas nitroreducens T	DSM 14399	WS (TUM)	oil-brine, Japan
Pseudomonas orientalis T	DSM 17489	DSMZ	spring water, Lebanon
Pseudomonas oryzihabitans	WS 5017	WS (TUM)	unknown, Germany
Pseudomonas oryzihabitans T	DSM 6835	DSMZ	rice paddy, Japan
Pseudomonas panacis	WS 4668	WS (TUM)	raw milk, Germany
Pseudomonas paralactis T	DSM 29164	DSMZ	raw milk, Germany
Pseudomonas peli T	DSM 17833	DSMZ	nitrifying enrichment culture, Belgium
Pseudomonas poae T	DSM 14936	DSMZ	phyllosphere of grasses, Germany
Pseudomonas protegens P3	**WS 5082**	WS (TUM)	raw milk, Germany
Pseudomonas protegens P3	**WS 5415**	WS (TUM)	raw milk, Germany
Pseudomonas proteolytica P1	**WS 5127**	WS (TUM)	raw milk, Germany
Pseudomonas proteolytica P1	**WS 5128**	WS (TUM)	raw milk, Germany
Pseudomonas psychrophila T	DSM 17535	DSMZ	coldroom for food storage, Japan

Species	Strain	Strain collection	Origin
Pseudomonas psychrotolerans T	DSM 15758	DSMZ	water under a dog's cage in a Medical Clinic for Small Animals, Austria
Pseudomonas rhodesiae T	DSM 14020	DSMZ	mineral water, France
Pseudomonas saxonica	DSM 108990	DSMZ	raw milk, Germany
Pseudomonas saxonica T	DSM 108989	DSMZ	raw milk, Germany
Pseudomonas soli T	DSM 28043	DSMZ	soil, Spain
***Pseudomonas* sp. 5** P3	**WS 5414**	WS (TUM)	raw milk, Germany
***Pseudomonas* sp. 6** P5	**WS 5071**	WS (TUM)	raw milk, Germany
***Pseudomonas* sp. 9** P1	**WS 5411**	WS (TUM)	raw milk, Germany
***Pseudomonas* sp. 9** P1	**WS 5412**	WS (TUM)	raw milk, Germany
***Pseudomonas* sp. 9** P1	**WS 5413**	WS (TUM)	raw milk, Germany
***Pseudomonas* sp. 10** P1	**WS 5059**	WS (TUM)	raw milk, Germany
***Pseudomonas* sp. 12** P2	**WS 5407**	WS (TUM)	raw milk, Germany
***Pseudomonas* sp. 12** P2	**DSM 29142**	DSMZ	raw milk, Germany
Pseudomonas stutzeri	WS 5018	WS (TUM)	environmental monitoring for pharmaceutical industry
Pseudomonas synxantha T	DSM 18928	DSMZ	cream, USA
Pseudomonas syringae	LMG 5141	DSMZ	*Cucumis sativus*, USA
Pseudomonas syringae subsp*. Syringae* T	DSM 10604	DSMZ	*Syringa vulgaris*, UK
Pseudomonas taetrolens T	DSM 21104	DSMZ	musty egg, unknown
Pseudomonas trivialis T	DSM 14937	DSMZ	phyllosphere of grasses, Germnay
Pseudomonas veronii	WS 5113	WS (TUM)	raw milk, Germany
Pseudomonas versuta T	DSM 101070	DSMZ	Soil, Antarctica, Lagoon Island
Pseudomonas weihenstehpanensis	DSM 29140	DSMZ	raw milk, Germany
Psychrobacter alimentarius	WS 5483	WS (TUM)	dairy product, Germany
Psychrobacter psychrophilus	WS 5484	WS (TUM)	air, Germany
Serratia liquefaciens	DSM 30065	DSMZ	raw milk, unkown
Staphylococcus agnetis	WS 5485	WS (TUM)	raw milk, Germany
Staphylococcus chromogenes	WS 5486	WS (TUM)	raw milk, Germany
Staphylococcus haemolyticus	WS 5488	WS (TUM)	raw milk, Germany
Staphylococcus sciuri	WS 5489	WS (TUM)	raw milk, Germany
Stenotrophomonas maltophilia	WS 5490	WS (TUM)	dairy product, Germany
Stenotrophomonas rhizophila	WS 5491	WS (TUM)	Fish delicacies, Germany
Streptococcus equinus	WS 5487	WS (TUM)	raw milk, Germany
Streptococcus mitis T	DSM 12643	DSMZ	oral cavity, human, unkown
Streptococcus pyogenes	DSM 2071	DSMZ	human isolate, unkown

T: type strains
P1-5: target strain of probe P1-P5

Table S2 Genome data of 61 *Pseudomonas* strains from NCBI used for the construction of *rpoB*- and *aprX*-phylogenetic trees und for primer and probe design

Strain	NCBI WGS ID	RefSeq/GenBank IDs
P. aeruginosa WS 5022	JAAQZH01	
P. asplenii ATCC 23835 T		LT629777.1
P. azotoformans LMG 21611 T		NZ_LT629702.1
P. brenneri WS 5091	JAAQZG01	
P. canadensis 2-92 T	AYTD01	
P. cedrina ssp. *cedrina* DSM 17516 T	MNPW01	
P. chlororaphis ssp. *chlororaphis* DSM 50083 T	VFIN01	
P. corrugata DSM 7228 T	LHVK01	
P. deceptionensis DSM 26521 T	JYKX01	
P. fluorescens WS 5037	JAAQZD01	
P. fluorescens DSM 50090 T	VFEP01	
P. fragi WS 5094	JAAQYX01	
P. fragi WS 5112	JAAQYV01	
P. fragi WS 5416	JAAQYQ01	
P. gessardii WS 5409	JAAQYP01	
P. gessardii WS 5049	JAAQYO01	
P. gessardii WS 5408	JAAQYN01	
P. graminis DSM 11363 T	FOHW01	
P. grimontii DSM 17515 T	VFES01	
P. haemolytica DSM 108987 T	VOIW01	
P. jessenii DSM 17150 T	NIWT01	
P. kilonensis DSM 13647 T	LHVH01	
P. koreensis DSM 16610 T	JAAQYM01	
P. lactis WS 5000	JAAQYJ01	
P. lactis WS 5404	JAAQYI01	
P. lactis WS 5405	JAAQYH01	
P. lactis DSM 29167 T	JYLO01	
P. libanensis DSM 17149 T	JYLH01	
P. lini DSM 16768 T	JYLB01	
P. lundensis WS 5024	JAAQYG01	
P. lundensis WS 5095	JAAQYC01	
P. lurida LMG 21995 T	PDJB01	
P. mandelii DSM 17967 T	VFIM01	
P. marginalis DSM 13124 T	VFEQ01	
P. mosselii DSM 17497 T	JHYW01	
P. mucidolens NBRC 103159 T	BDAH01	
P. orientalis DSM 17489 T	JYLM01	
P. panacis WS 4668	JAARME01	
P. paralactis DSM 29164 T	JYLN01	
P. poae DSM 14936 T	JYLI01	
P. protegens WS 5082	JAARMD01	
P. protegens WS 5415	JAAQXO01	
P. proteolytica WS 5127	JAAQXK01	
P. proteolytica WS 5128	JAAQXJ01	
P. rhodesiae WS 4669	JAAQXF01	
P. rhodesiae DSM 14020 T	VFEU01	
P. soli LMG 27941 T	FOEQ01	
Pseudomonas sp. 5 WS 5414	JAAQWZ01	
Pseudomonas sp. 6 WS 5071	JAAQWY01	
Pseudomonas sp. 9 WS 5411	JAARMC01	
Pseudomonas sp. 9 WS 5412	JAARMB01	
Pseudomonas sp. 9 WS 5413	JAAQWR01	
Pseudomonas sp. 10 WS 5059	JAAQWO01	
Pseudomonas sp. 12 DSM 29142	JYLP01	
Pseudomonas sp. 12 WS 5407	JAAQWJ01	

Strain	NCBI WGS ID	RefSeq/GenBank IDs
P. synxantha DSM 18928 T	JYLJ01	
P. syringae ssp. *syringae* DSM 10604 T	JALK01	
P. trivialis DSM 14937 T	JYLK01	
P. veronii WS 5113	JAAQWD01	
P. versuta L10.10 T		NZ_CP012676.1
P. weihenstephanensis DSM 29140	JYLG01	

T : type strain

Table S3 Composition of six different DNA-pools used as templates in single- and multiplex qPCR. Every DNA-pool (pool 1-6) contains equal DNA amounts of five different strains, each representing a target for one species-specific probe (P1-P5)

Probe	Species	Strain	Pool 1	Pool 2	Pool3	Pool 4	Pool 5	Pool 6
**P1**	*P. proteolytica*	WS 5127	X					
	P. proteolytica	WS 5128		X				
	P. gessardii	WS 5408			X			
	P. gessardii	WS 5049				X		
	Pseudomonas sp. 9	WS 5413					X	
	Pseudomonas sp. 9	WS 5411						X
**P2**	*P. lactis*	WS 5404	X					
	P. lactis	DSM 29167		X				
	P. fluorescens	WS 5037			X			
	P. fluorescens	DSM 50090				X		
	Pseudomonas sp. 12	WS 5407					X	
	Pseudomonas sp. 12	DSM 29142						X
**P3**	*Pseudomonas* sp. 5	WS 5414	X		X		X	
	P. protegens	WS 5082		X		X		X
**P4**	*P. fragi*	WS 5416	X		X		X	
	P. fragi	WS 5112		X		X		X
**P5**	*P. lundensis*	WS 5095	X		X		X	
	P. lundensis	WS 5024		X		X		X

Table S4 Cq-values from multiplex qPCR assays, applying the six *Pseudomonas* hydrolysis probes (P1-P6) and DNA (0.1-0.2 ng/µl final concentration) from 49 *Pseudomonas* strains from 45 non-target species and isolates of 40 other bacterial species. Mean values of two technical replicates per measurement are shown. Hyphens (-) represent no signal in qPCR. C_q-values above the defined threshold of 33 were considered as not quantifiable and are given in brackets

Species and strain	P1	P2	P3	P4	P5	P6
**Non-target pseudomonads**						
Pseudomonas aeruginosa WS 5022	-	-	-	-	-	22.69
Pseudomonas alcaligenes WS 5013	-	-	-	-	-	20.82
Pseudomonas asplenii DSM 17133 T	-	-	-	-	-	19.13
Pseudomonas brenneri WS 5091	-	-	-	-	-	18.51
Pseudomonas canadensis LMG 28499 T	-	-	-	-	-	20.08
Pseudomonas cedrina DSM 17516 T	-	-	-	-	-	20.35
Pseudomonas chengduensis DSM 26382 T	-	-	-	-	-	22.63

Species and strain	P1	P2	P3	P4	P5	P6
Pseudomonas chlororaphis DSM 50083 T	-	-	-	-	-	20.60
Pseudomonas corrugata DSM 7228 T	-	-	-	-	-	19.92
Pseudomonas deceptionensis DSM 26521 T	-	-	-	-	-	19.93
Pseudomonas endophytica WS 5129	-	-	-	-	-	19.97
Pseudomonas extremaustralis DSM 17835 T	-	-	-	-	-	18.64
Pseudomonas graminis DSM 100941	-	-	-	-	-	22.63
Pseudomonas grimontii DSM 17515 T	32.13	-	-	-	-	18.79
Pseudomonas haemolytica DSM 108987 T	-	26.00	-	-	-	20.43
Pseudomonas helleri DSM 29165 T	-	-	-	-	-	20.00
Pseudomonas jessenii DSM10315	-	-	-	-	-	18.24
Pseudomonas jessenii WS 5418	-	-	-	-	-	19.09
Pseudomonas kilonensis DSM 13647 T	-	-	-	-	-	20.05
Pseudomonas koreensis DSM 16610 T	-	-	-	-	-	19.01
Pseudomonas libanensis DSM 17149 T	-	-	-	-	-	20.07
Pseudomonas lini DSM 16768 T	-	-	-	-	-	18.64
Pseudomonas mandelii DSM 17967 T	-	-	-	-	-	17.45
Pseudomonas marginalis DSM 13124	32.12	-	-	-	-	18.73
Pseudomonas mosselii DSM 17497 T	-	-	-	-	-	21.81
Pseudomonas mucidolens WS 5456	-	-	-	-	-	20.32
Pseudomonas nitroreducens DSM 14399 T	-	-	-	-	-	21.38
Pseudomonas orientalis DSM 17489 T	-	24.45	-	-	-	18.81
Pseudomonas oryzihabitans DSM 6835 T	-	-	-	-	-	18.70
Pseudomonas oryzihabitans WS 5017	-	-	-	-	-	18.62
Pseudomonas panacis WS 4668	-	-	-	-	-	20.16
Pseudomonas paralactis DSM 29164 T	-	24.44	-	-	-	18.74
Pseudomonas peli DSM 17833 T	-	-	-	-	-	20.06
Pseudomonas poae DSM 14936 T	-	(33.31)	-	-	-	19.77
Pseudomonas psychrophila DSM 17535 T	-	-	-	-	-	18.14
Pseudomonas psychrotolerans DSM 15758 T	-	-	-	-	-	18.49
Pseudomonas rhodesiae DSM 14020 T	-	-	-	-	-	20.62
Pseudomonas saxonica DSM 108989 T	-	-	-	-	-	19.97
Pseudomonas saxonica DSM 108990	-	-	-	-	-	19.69
Pseudomonas soli DSM 28043 T	-	-	-	-	-	22.53
Pseudomonas stutzeri WS 5018	-	-	-	-	-	27.13
Pseudomonas synxantha DSM 18928 T	-	27.61	-	-	-	19.82
Pseudomonas syringae LMG 5141	-	-	-	-	-	20.19
Pseudomonas syringae subsp. *Syringae* DSM 10604 T	-	-	-	-	-	20.29
Pseudomonas taetrolens DSM 21104 T	-	-	-	-	-	18.02
Pseudomonas trivialis DSM 14937 T	(33.27)	(34.19)	-	-	-	18.21
Pseudomonas veronii WS 5113	-	-	-	-	-	19.72
Pseudomonas versuta DSM 101070 T	-	-	-	-	-	19.96
Pseudomonas weihenstehpanensis DSM 29140	-	-	-	-	-	19.7

Species and strain	P1	P2	P3	P4	P5	P6
**Other bacteria**						
Acinetobacter guillouiae WS 5457	-	-	-	-	-	(33.70)
Acinetobacter johnsonii WS 5458	-	-	-	-	-	(33.84)
Acinetobacter junii WS 5459	(34.87)	-	-	-	-	-
Aerococcus viridans WS 5460	(33.23)	-	-	-	-	-
Arthrobacter casei WS 5462	-	-	-	-	-	-
Arthrobacter ilicis WS 5461	-	-	-	-	-	-
Bacillus licheniformis DSM 8785	-	-	-	-	-	-
Bifidobacterium longum DSM 20219 [T]	-	-	-	-	-	-
Brevibacterium aurantiacum WS 5463	-	-	-	-	-	-
Brevibacterium pityocampae WS 5464	-	-	-	-	-	(33.99)
Chryseobacterium haifense WS 5465	-	-	-	-	-	-
Citrobacter freundii WS 5466	-	-	-	-	-	**30.96**
Corynebacterium comporealensis WS 5467	-	-	-	-	-	-
Corynebacterium stationis WS 5469	-	-	-	-	-	-
Delftia lacustris WS 5470	(33.31)	-	-	-	-	**32.91**
Enhydrobacter aerosaccus WS 5471	-	-	-	-	-	(33.46)
Enterobacter cowanii WS 5472	-	-	-	-	-	-
Enterococcus durans WS 5473	-	-	-	-	-	-
Enterococcus italicus WS 5474	-	-	-	-	-	-
Hafnia alvei DSM 30098	-	-	-	-	-	**32.47**
Kocuria kristinae WS 5475	-	-	-	-	-	-
Lactobacillus paracasei WS 5476	-	-	-	-	-	-
Lactococcus garvieae WS 5478	-	-	-	-	-	-
Lactococcus lactis WS 5479	-	-	-	-	-	-
Lactococcus raffinolactis DSM 20443 [T]	-	-	-	-	-	-
Leuconostoc lactis WS 5480	(33.90)	-	-	-	-	-
Microbacterium hominis WS 5481	-	-	-	-	-	-
Pseudoalteromonas haloplanktis WS 5482	-	-	-	-	-	**29.61**
Psychrobacter alimentarius WS 5483	-	-	-	-	-	-
Psychrobacter psychrophilus WS 5484	(34.37)	-	-	-	-	-
Serratia liquefaciens DSM 30065	-	-	-	-	-	**32.76**
Staphylococcus agnetis WS 5485	-	-	-	-	-	-
Staphylococcus chromogenes WS 5486	(34.29)	-	-	-	-	-
Staphylococcus haemolyticus WS 5488	-	-	-	-	-	-
Staphylococcus sciuri WS 5489	(34.87)	-	-	-	-	-
Stenotrophomonas maltophilia WS 5490	-	-	-	-	-	-
Stenotrophomonas rhizophila WS 5491	-	-	-	-	-	-
Streptococcus equinus WS 5487	(34.58)	-	-	-	-	-
Streptococcus mitis DSM 12643 [T]	-	-	-	-	-	-
Streptococcus pyogenes DSM 2071	**32.91**	-	-	-	-	-

[T]: type strains

Table S5 Total- and *Pseudomonas* cell counts of 60 industrial raw milk samples, determined by cultivation on TSA and CFC agar, respectively. ND: not determinded

Raw milk sample	Total cell count	*Pseudomonas* cell count	P*seudomonas* / total count [%]
1	1.10E+07	9.50E+06	86.7
2	>1.00E+08	2.00E+07	ND
3	1.20E+07	5.95E+06	49.6
4	2.00E+07	2.00E+07	100.0
5	>1.00E+08	1.07E+07	ND
6	>1.00E+08	1.16E+07	ND
7	7.00E+06	5.00E+06	71.4
8	2.00E+07	3.59E+06	18.0
9	6.51E+05	5.05E+05	77.5
10	3.17E+06	1.46E+06	46.1
11	1.29E+06	8.20E+05	63.6
12	6.23E+06	2.84E+06	45.6
13	>1.00E+08	3.24E+06	ND
14	4.30E+05	5.10E+05	118.6
15	1.75E+05	2.07E+05	118.1
16	>1.00E+05	3.86E+05	ND
17	6.88E+05	4.46E+05	64.9
18	1.87E+05	1.59E+05	85.0
19	3.80E+04	2.84E+04	74.6
20	1.36E+05	5.44E+04	40.0
21	1.92E+06	2.62E+05	13.6
22	9.50E+05	4.68E+05	49.3
23	1.60E+04	3.00E+02	1.9
24	9.91E+04	8.80E+04	88.8
25	6.00E+04	5.80E+04	96.7
26	1.23E+05	3.55E+04	29.0
27	5.55E+05	4.53E+04	8.2
28	1.92E+05	1.96E+05	102.4
29	4.66E+05	3.11E+05	66.7
30	1.10E+05	6.71E+04	61.0
31	1.16E+06	2.28E+05	19.7
32	2.72E+05	1.84E+05	67.6
33	2.73E+05	1.09E+05	40.0
34	1.25E+05	8.10E+04	64.8
35	1.07E+05	1.87E+04	17.5
36	8.30E+04	2.25E+04	27.1
37	9.82E+04	2.04E+04	20.8
38	3.00E+04	3.20E+03	10.7
39	1.40E+05	2.09E+04	14.9
40	3.20E+04	1.20E+03	3.8
41	6.70E+04	1.79E+04	26.7

Raw milk sample	Total cell count	*Pseudomonas* cell count	P*seudomonas* / total count [%]
42	2.06E+05	1.35E+04	6.6
43	2.00E+06	3.21E+04	1.6
44	2.30E+04	8.00E+02	3.5
45	3.45E+05	8.10E+03	2.4
46	1.07E+05	1.18E+04	11.0
47	2.82E+04	4.20E+03	14.9
48	1.80E+04	3.00E+03	16.7
49	6.20E+04	2.75E+04	44.3
50	5.40E+04	5.60E+03	10.4
51	2.30E+04	5.55E+03	24.1
52	2.40E+04	2.60E+03	10.8
53	3.00E+04	4.50E+03	15.0
54	2.30E+04	1.50E+03	6.5
55	2.50E+04	9.00E+02	3.6
56	2.28E+05	9.18E+03	4.0
57	2.00E+04	6.00E+02	3.0
58	1.60E+04	2.20E+03	13.8
59	2.90E+04	2.10E+03	7.2
60	6.30E+04	6.00E+02	1.0
